# Dr. Charles S. Barney

**Published:** 1914-08

**Authors:** 


Dr. Charles S. Barney, Albany, 1883, died at his home in Milford, May 26, aged 55.

